# Guided Multispectral Optoacoustic Tomography for 3D Imaging of the Murine Colon

**DOI:** 10.1002/advs.202413434

**Published:** 2025-01-21

**Authors:** Adrian Buehler, Emma L. Brown, Markus Eckstein, Oana‐Maria Thoma, Felix Wachter, Henriette Mandelbaum, Petra Ludwig, Merle Claßen, Mariam‐Eleni Oraiopoulou, Ulrich Rother, Markus F. Neurath, Joachim Woelfle, Maximilian J. Waldner, Oliver Friedrich, Ferdinand Knieling, Sarah E. Bohndiek, Adrian P. Regensburger

**Affiliations:** ^1^ Department of Pediatrics and Adolescent Medicine University Hospital Erlangen Friedrich‐Alexander‐Universität Erlangen‐Nürnberg 91054 Erlangen Germany; ^2^ Department of Physics and Cancer Research UK Cambridge Institute University of Cambridge Cambridge CB2 0RE UK; ^3^ Institute of Pathology University Hospital Erlangen Friedrich‐Alexander‐Universität Erlangen‐Nürnberg 91054 Erlangen Germany; ^4^ Department of Medicine 1 University Hospital Erlangen Friedrich‐Alexander‐Universität Erlangen‐Nürnberg 91054 Erlangen Germany; ^5^ Deutsches Zentrum Immuntherapie DZI, University Hospital Erlangen 91054 Erlangen Germany; ^6^ Department of Vascular Surgery University Hospital Erlangen Friedrich‐Alexander‐Universität Erlangen‐Nürnberg 91054 Erlangen Germany; ^7^ Institute of Medical Biotechnology Friedrich‐Alexander‐Universität Erlangen‐Nürnberg 91052 Erlangen Germany

**Keywords:** dextran sodium sulfate induced colitis, inflammatory bowel disease, multispectral optoacoustic tomography, murine acute colitis, optoacoustic imaging, photoacoustic imaging

## Abstract

Multispectral optoacoustic tomography is a promising medical imaging modality that combines light and sound to provide molecular imaging information at depths of several centimeters, based on the optical absorption of endogenous chromophores, such as hemoglobin. Assessment of inflammatory bowel disease has emerged as a promising clinical application of optoacoustic tomography. In this context, preclinical studies in animal models are essential to identify novel disease‐specific imaging biomarkers and understand findings from emerging clinical pilot studies, however to‐date, these studies have been limited by the precise identification of the bowel wall. Herein, a transrectal‐absorber guide is applied, serving as a high‐contrast landmark for 3D optoacoustic tomography of the colon. This study shows that guided multispectral optoacoustic tomography is able to measure changes in blood oxygenation status over the course of acute, chemically‐induced colitis in mice and correlates with standard disease activity scores. This novel approach depicts intestinal hemoglobin composition non‐invasively during murine inflammation. These results underscore the potential for optoacoustic imaging in translational inflammatory bowel disease research.

## Graphical abstract

Multispectral optoacoustic tomography (MSOT) is about to enter clinical practice for inflammatory bowel disease (IBD) assessment. In this study, a transrectal absorber guide (TAG) is introduced for precise localization and 3D imaging of the colon wall of mice for preclinical IBD research. Subsequently, a chemically induced murine colitis model is used to evaluate TAG‐MSOT functional imaging biomarkers.

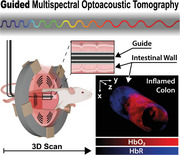

## 1 Introduction

Inflammatory bowel diseases (IBD) are a health burden with rising incidence.^[^ ^1^ ^]^ With increasing incidence, there are also more varied and often more difficult‐to‐treat courses of the disease. New therapies targeting different aspects of the inflammatory pathway appear promising, and such therapeutics require early stage evaluation in preclinical small animal studies before proceeding to first‐in‐human testing.^[^ ^2^ ^]^ Due to the multifactorial genesis of IBD in humans, several mouse models for IBD have been developed, each mimicking different aspects of the disease.^[^ ^3^ ^]^ Monitoring of disease status in these mouse models is typically achieved by some combination of small animal endoscopy, directly at the site of interest, depth‐resolved methods such as ultrasound, or with tomographic imaging through magnetic resonance or computed tomography technologies.^[^ ^4^ ^]^

Key biomarkers of interest for evaluating disease activity and treatment response are the transmural microvasculature hemoglobin concentration and oxygenation status, which are altered in IBD patients.^[^ ^5^ ^]^ While existing imaging technologies go some way toward interrogating these biomarkers, there remain limitations that could be addressed through the introduction of optoacoustic technologies.^[^ ^6^ ^]^ Mesoscopic optoacoustic imaging provides high resolution (≈20 microns in‐plane) at several millimeters imaging depth from a single viewpoint, providing direct analysis of small intramural vessels within the field of view, quantifying their respective networks as well as blood volume. Two main limitations of mesoscopic optoacoustic imaging include its typical use of a single wavelength, which restricts oxygenation information, and its relatively superficial imaging of only one region of the colon wall.^[^ ^7^ ^]^

Multispectral approaches that acquire images at multiple wavelengths are able to differentiate between deoxygenated and oxygenated hemoglobin based on their distinct photoacoustic properties.^[^ ^8^ ^]^ Furthermore, multispectral optoacoustic tomography (MSOT) enables imaging at depths of several centimeters, albeit at lower resolution of ≈100 microns. A prior study with MSOT revealed elevated levels of oxygenated hemoglobin in a mouse model of bacterial‐induced colitis that correlated well with disease activity.^[^ ^9^ ^]^ Furthermore in the clinic, handheld MSOT with integrated ultrasound guidance has been applied in IBD patients.^[^ ^10^ ^]^ Here, increased levels of hemoglobin correlated well with the disease activity in patients with Crohn`s disease.^[^ ^11^ ^]^ In pediatric patients, increased deoxygenated hemoglobin was measured in Crohn`s disease while increased oxygenated hemoglobin was measured in patients with ulcerative colitis.^[^ ^12^ ^]^

Application of MSOT for the longitudinal study of colitis of the colon wall in animal models has thus far been limited by challenges in identifying the precise position of the colon wall at depth in living subjects, restricting our capabilities to better understand the underpinning biology of clinical findings with MSOT biomarkers and to evaluate the impact of new therapeutics on disease activity measured through these biomarkers. To improve the status of translational research in IBD, we now established a transrectal absorber guide for MSOT imaging of the murine colon and tested this approach in a mouse model of chemically‐induced colitis to accelerate translational optoacoustic imaging studies in IBD research and support clinical application of optoacoustic technologies.

## 2 Results

### 2.1 Development of a Guided Tomographic Imaging Approach

**Figure 1:**
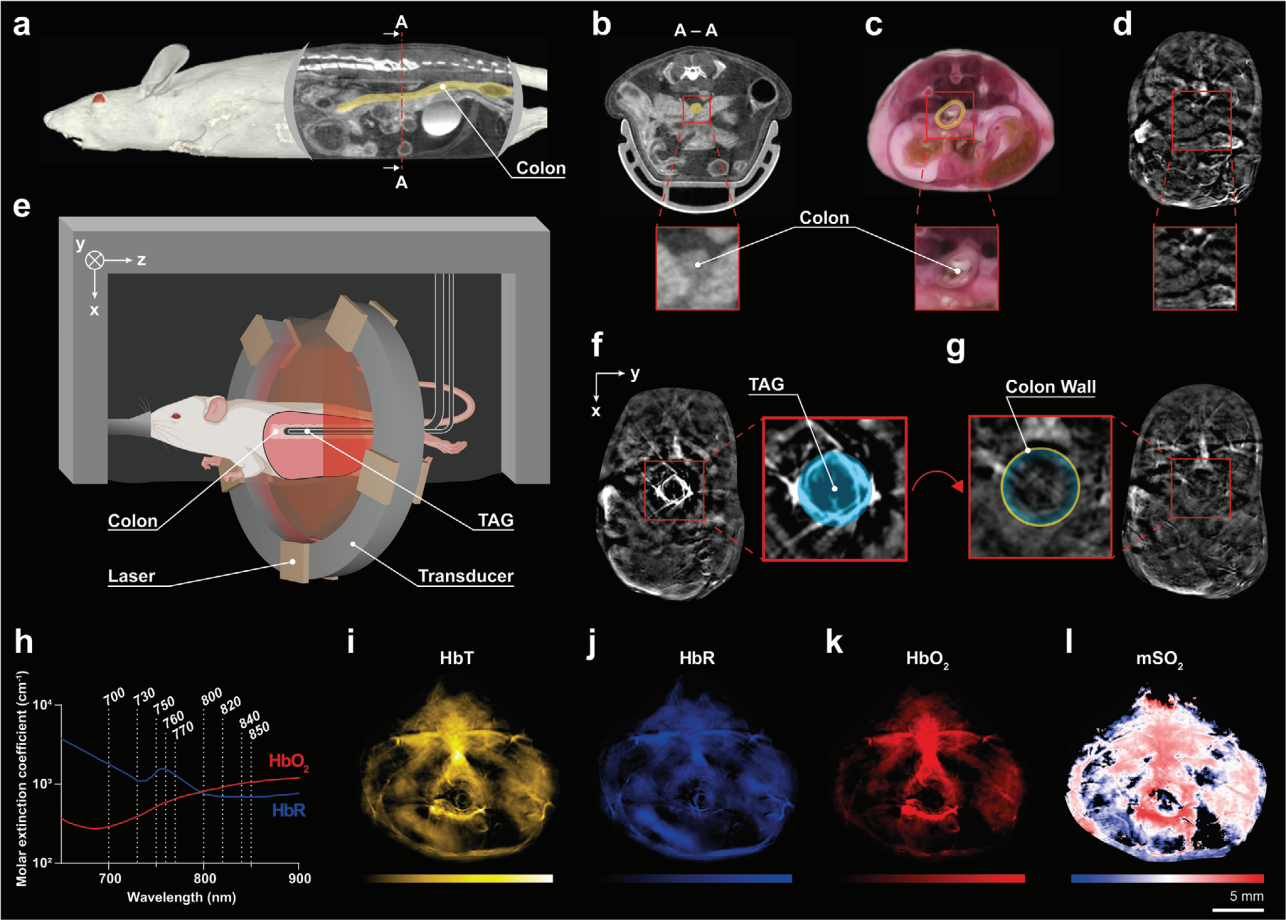
TAG‐MSOT imaging of hemoglobin composition of the murine colon. a) The linear alignment of the colon from the descending colon to the rectum and up to the anus is depicted in the sagittal plane of a µCT scan.^[^ ^29^ ^]^ The central position of the colon in the abdomen of the mouse is visualized in the coronal plane of the same scan in b) and in a cryosection in c).^[^ ^30^ ^]^ d) In contrast, MSOT alone cannot reliably identify the colon (high‐pass filtered 800 nm signal for illustration only). Therefore, we developed and implemented an imaging protocol using a TAG placed inside the mouse during imaging as shown in e). By performing two successive scans, one with and one without a contrast agent, the TAG can be reliably identified in one image f) and allows for the quantification of the adjacent bowel wall in the corresponding second image g) without artifacts caused by the contrast. h) Imaging at nine different wavelengths sequentially allows for the calculation of oxygenated and deoxygenated hemoglobin based on their characteristic absorption spectra. i–l) Subsequent spectral unmixing of total hemoglobin (HbT), deoxygenated hemoglobin (HbR), oxygenated hemoglobin (HbO_2_), and blood saturation (mSO_2_) in the intestinal wall are shown for exemplary MSOT data of a healthy mouse.

The colon presents a difficult target for optoacoustic imaging because of the small dimensions of the intestinal wall and the central position of the colon (**Figure** **1**a–d). The use of a transrectal absorber guide (TAG) (Figure S1, Supporting Information) provides an innovative solution for optoacoustic imaging of the murine colon, previously introduced in combination with raster‐scanning optoacoustic mesoscopy.^[^ ^7^ ^]^ In brief, the TAG is a passive rectal probe placed into the colon during imaging, serving as a high‐contrast landmark (Figure 1e). The TAG design provides a stable backbone to support the colon during imaging and facilitates the exchange of contrast fluid without repositioning the animal, enabling acquisition of images both with and without the high‐contrast fiducial. The TAG's simple geometry and high contrast are identified in one scan, allowing for the quantification of the adjacent colon wall in a corresponding second scan without the presence of the contrast media, avoiding potential saturation effects (Figure 1f,g). In this work, the TAG was used in combination with a commercially available preclinical MSOT system. A total of nine different wavelengths between 700 and 850 nm were used for image acquisition, allowing the unmixing of oxygenated hemoglobin (HbO_2_) and deoxygenated hemoglobin (HbR) by linear regression, and the subsequent calculation of the total hemoglobin (HbT) and the oxygen saturation (mSO_2_) (Figure 1h,l).

### 2.2 Defining the Optimal Imaging Volume

Next, a chemically‐induced colitis based on dextran sodium sulfate (DSS) administration was used to emulate acute colitis in a mouse model. To reduce animal burden, all animals were also used in a parallel study using mesoscopic optoacoustic imaging,^[^ ^7^ ^]^ with validation of the disease burden of the DSS model by clinical, endoscopic, and histological scoring in *Buehler* et al.^[^ ^7a^ ^]^ To induce mild acute colitis in mice (n = 10), a concentration of 5% DSS dissolved in drinking water was administered for one week. TAG‐MSOT imaging was performed before and two days after the end of DSS administration, where the point of highest inflammation is expected. A sham group (n = 5) received no DSS and was used to test potential influence of the imaging protocol on the data.

**Figure 2:**
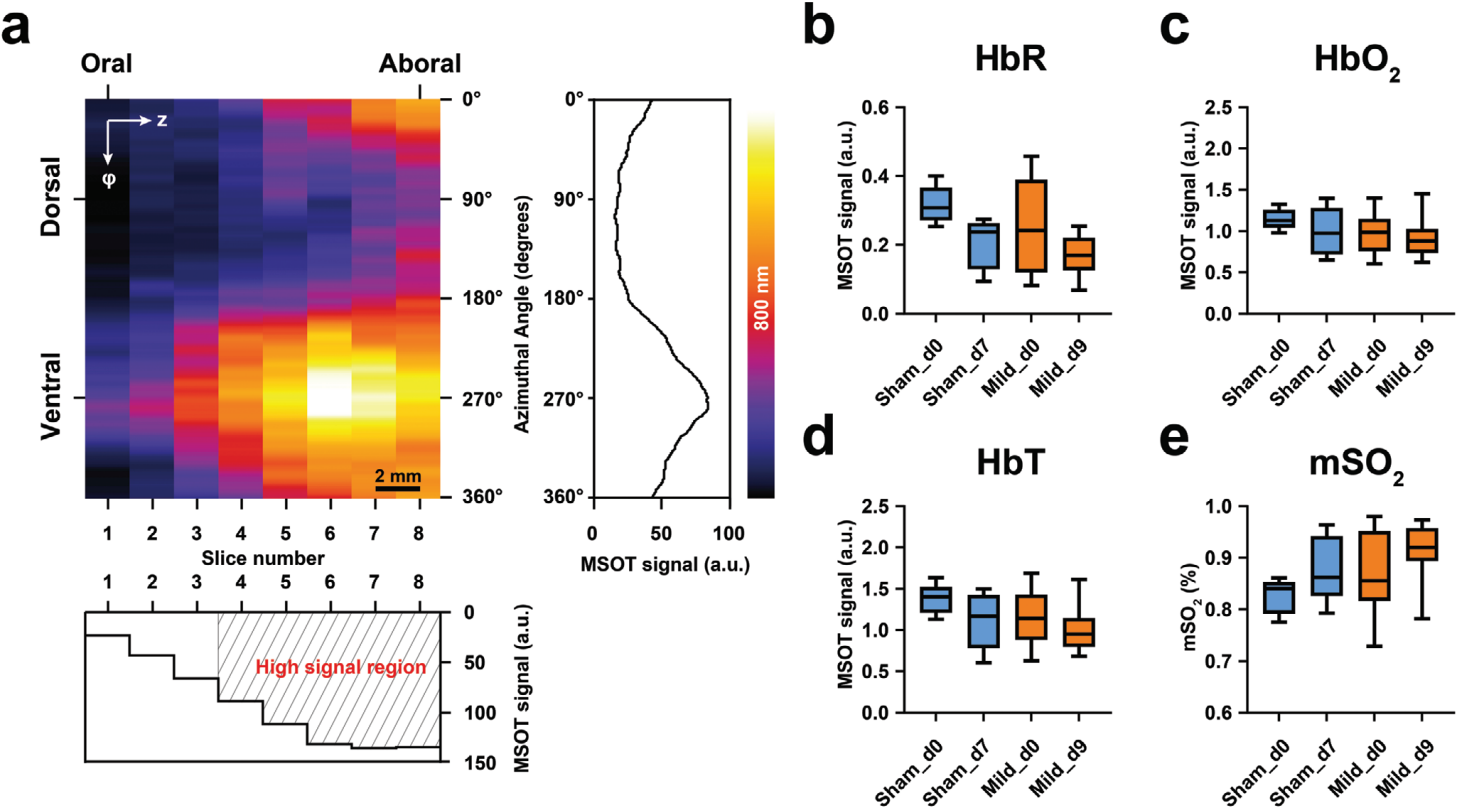
TAG‐MSOT of mild acute colitis in mice. Mice (n = 10) were imaged before (Mild_d0) and two days after (Mild_d9) a one‐week cycle of 5% DSS administration to induce mild colitis. A sham control group (n = 5) was imaged two times within seven days (Sham_d0 and Sham_d7) while receiving untreated sterile drinking water only. a) Average intensity projections in radial direction of polar‐transformed (pt) colon walls (CW) (see Figure S2, Supporting Information) of Sham_d0, Sham_d7, Mild_d0, and Mild_d9 were averaged to reveal a signal quality gradient from oral to aboral at an isosbestic point of oxygenated (HbO_2_) and deoxygenated (HbR) hemoglobin (800 nm) (for individual CW_pt_ see Figures S3–S6, Supporting Information). Therefore, quantification of the MSOT parameters is based on an aboral subsection of high signal intensity (slice numbers 4 to 8): b) HbR, c) HbO_2_, d) total hemoglobin (HbT), and e) blood saturation (mSO_2_) shows no significant difference (*P* > 0.05) between the groups in mild colitis. The data is presented as box plots (showing the median, 25th, and 75th percentiles, with whiskers representing the minimum and maximum values). Statistical analyses were performed using one‐way ANOVA with Šidák's correction (b–e).

Tomographic images were visually inspected to determine the region in which the contrast‐filled TAG could be clearly and confidently identified for region‐of‐interest drawing. The contrast‐filled TAG could only be reliably identified between the thoracic cage and the pelvic region. To enhance interpretation of the circumferential data, radial projections of polar‐transformed TAG‐MSOT data were made (Figure S2, Supporting Information). An average intensity projection of scans without contrast of the sham and mild colitis study at 800 nm revealed a heterogenous signal quality distribution in the measurable colon wall (**Figure** **2**a). Furthermore, the signal is not isotropic around the TAG‐axis. Based on this heterogeneous distribution, the quantification was defined to an aboral 8 mm segment of the colon, corresponding to the analysis of five consecutive slices. With an optimal image volume identified, MSOT biomarkers were quantified; these findings revealed a slight decrease in HbR and a successive minor increase in mSO_2_, but the mild inflammation induced in this first cycle did not produce any significant changes (*P* > 0.05) of the MSOT signal (Figure 2b–e; Figures S3 and S4, Supporting Information).

### 2.3 TAG‐MSOT Enables Analysis of Hemoglobin Composition in Severe Colitis

**Figure 3:**
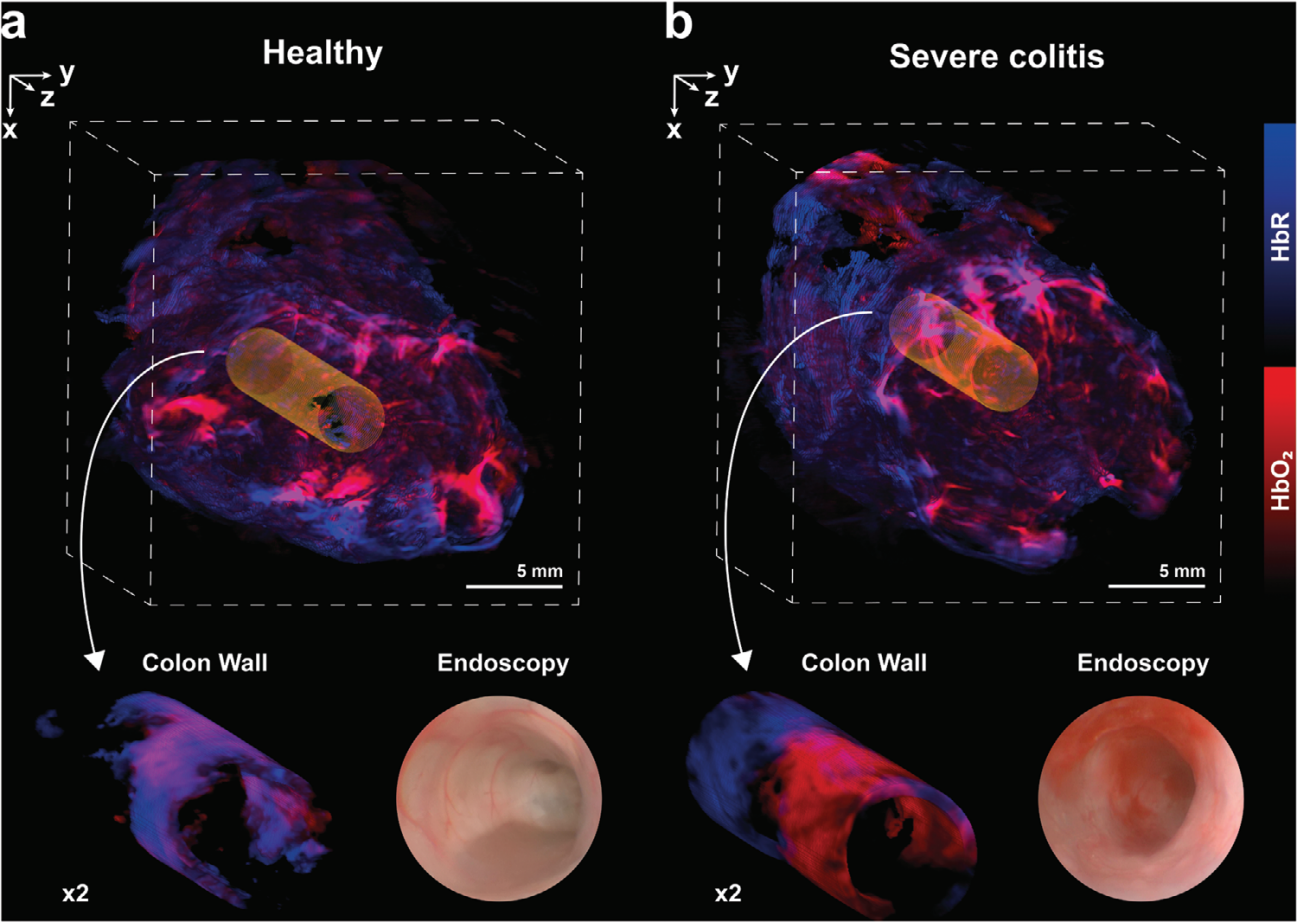
TAG‐MSOT for 3D assessment of inflammation of the murine colon. TAG‐MSOT shows visible changes in oxygenated and deoxygenated hemoglobin content in the colon wall during inflammation. An exemplary optoacoustic image of a mouse abdomen in 3D is shown a) before and b) after induction of severe colitis with two cycles of DSS administration (Video S1, Supporting Information). The orientation of the volumes corresponds to the coordinate system in Figure 1e. The colon with the respective optoacoustic signals for oxygenated and deoxygenated hemoglobin are cropped for visualization of changes during acute severe colitis. The corresponding endoscopic images of the colon are displayed. The depicted 3D rendering benefits from a high sampling rate of 0.5 mm along the Z‐axis of water‐filled TAG images. Precise quantification, however, was performed exclusively on reconstructions derived from slices with matching contrast‐filled TAG images.

**Figure 4:**
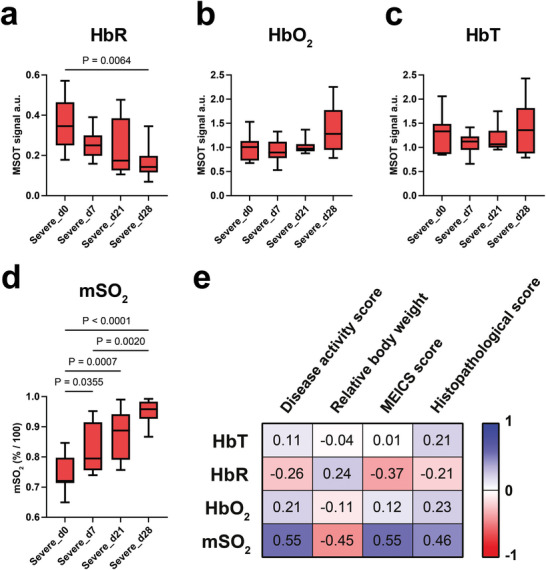
TAG‐ MSOT hemoglobin parameters for the assessment of severe colitis in mice. Severe colitis was induced in mice (n = 10) by administering two one‐week cycles of DSS with a two‐week healing phase in between. A first cycle of 3% DSS induced mild colitis (Severe_d7), while a second cycle of 5% DSS induced severe colitis (Severe_d28). At each timepoint, TAG‐MSOT was performed and the images were unmixed for a) deoxygenated hemoglobin (HbR) and b) oxygenated hemoglobin (HbO_2_). Subsequently, c) the total hemoglobin signal (HbT) and d) blood saturation (mSO_2_) were calculated. The data is presented as box plots (showing the median, 25th, and 75th percentiles, with whiskers representing the minimum and maximum values). Statistical analyses were performed using the Kruskal‐Wallis test with Dunn's correction (a–c) and one‐way ANOVA with Šidák's correction (d). Non‐significant results (*P* > 0.05) are not displayed in the graphs. e) Spearman correlation of mild and severe colitis groups for total hemoglobin (HbT), deoxygenated hemoglobin (HbR), oxygenated hemoglobin (HbO_2_), and blood saturation (mSO_2_) with disease activity score, relative body weight, endoscopic score (MEICS), and histopathological score are depicted as a correlation matrix. The disease activity score, relative body weight, endoscopic score (MEICS), and histopathological score of all studies can be found in *Buehler* et al.^[^ ^7a^ ^]^

Next, we induced a severe colitis in mice (n = 10) by administration of two cycles of DSS. Severe inflammation could be observed in MSOT images after spectral unmixing for hemoglobin and was also confirmed by health monitoring, with histology, endoscopy, and *ex vivo* colon length measurements (**Figure** **3**). Here, during the first cycle, a concentration of 3% DSS in drinking water was used to induce mild colitis. Analogous to day 9 of the mild colitis group, a slight increase in mSO_2_ was measured (d0 vs d7: 0.7419 ± 0.0598 a.u. vs 0.8235 ± 0.0811 a.u., P = 0.0355). HbO_2_ (d0 vs d7: 1.001 ± 0.2599 a.u. vs 0.9224 ± 0.2557 a.u., *P* > 0.9999) and HbR (d0 versus d7: 0.3627 ± 0.1300 a.u. vs 0.2553 ± 0.0717 a.u., P = 0.9606) did not change significantly but their trends were in line with the significant finding in mSO_2_ (**Figure** **4**); this is consistent with prior work where the ratiometric assessment of oxygenation has been found to be more robust than the individually unmixed parameters.^[^ ^13^ ^]^

After a two‐week healing period, a second cycle of 5% DSS was administered to induce severe colitis. Interestingly, the HbR signal (d0 vs d21: 0.3627 ± 0.1300 a.u. vs 0.2340 ± 0.1455 a.u., P = 0.1160) and the mSO_2_ (d0 vs d21: 0.7419 ± 0.0598 a.u. vs 0.8772 ± 0.0808 a.u., P = 0.0007) did not return to baseline levels after this healing period. At the end of the second cycle, severe inflammation manifested itself as a statistically non‐significant increase in HbO_2_ (d0 vs d28: 1.001 ± 0.2599 a.u. vs 1.363 ± 0.4966 a.u., P = 0.6550), a decrease in HbR (d0 vs d28: 0.3627 ± 0.1300 a.u. vs 0.1670 ± 0.0836 a.u., P = 0.0064), and an increase in mSO_2_ (d0 vs d28: 0.7419 ± 0.0598 a.u. vs 0.9492 ± 0.0407 a.u., *P* < 0.0001), showing functional differences and sensitivity of MSOT to vascular function (Figure 4; Figures S5 and S6, Supporting Information). Notably, as in the mild colitis study, the mean HbT signal did not change significantly during the disease progression, so the tomographic measurement was found to be less sensitive to changes in vascular density (Figure S7, Supporting Information). In mild and severe colitis, correlation between MSOT hemoglobin parameters and disease scores showed greatest correlation between mSO_2_ and disease activity score (r_s_ = 0.55, *P* <0.0001), as well as between the endoscopic (MEICS) score (r_s_ = 0.55, *P* < 0.0001) (Figure 4).

## 3 Discussion

In this work, a transrectal absorber guide (TAG) for preclinical multispectral optoacoustic tomography (MSOT) was implemented, allowing precise localization and 3D imaging of the colon wall and the surrounding tissue for quantification of oxygenated and deoxygenated hemoglobin and the respective oxygenation status. In a study of severe acute colitis, changes of the hemoglobin composition as sign of acute inflammation could be decoded.

TAG‐MSOT enabled precise localization of the colon for visualization and quantification of optoacoustic signals, confirming previous findings with mesoscopic optoacoustic imaging, referred to as TAG‐RSOM.^[^ ^7^ ^]^ TAG‐RSOM enables visualization of the microvasculature and the detection of mild signs of inflammation in models of acute colitis, even before tissue alterations detectable by histological analysis occur. In contrast, TAG‐MSOT offers a higher imaging depth, facilitating the assessment of the complete colon with the surrounding tissue and the analysis of vascular function based on changes in hemoglobin oxygenation. Since acoustic wave attenuation is frequency‐dependent, the spatial resolution can roughly be approximated as 1/200 of the imaging depth.^[^ ^14^ ^]^ TAG‐MSOT achieves greater imaging depth than TAG‐RSOM by utilizing ultrasound transduces with a center frequency an order of magnitude lower. Consequently, sub‐resolution colonic microvasculature cannot be resolved structurally but instead detected by the constructive optoacoustic signal interference, enabling functional imaging.^[^ ^15^ ^]^ We demonstrated that a chemically induced colitis leads to decreased deoxygenated hemoglobin levels, while the transmural oxygen saturation significantly increased. A significant change in total hemoglobin content was not observed, as might have been expected given prior TAG‐RSOM findings, however, the quantification of HbT with MSOT is less reliable than mSO_2_, which could explain this discrepancy.^[^ ^13^ ^]^ While TAG‐RSOM enables structural imaging of early inflammation of the murine colon, clinical translation of this high‐resolution OAI approach is limited by its endoscopic application.^[^ ^16^ ^]^ In contrast, TAG‐MSOT offers functional imaging with immediate translational application to clinical IBD research.

Considering the biological finding of elevated oxygenation observed by TAG‐MSOT, a potential explanation is the elevated epithelial oxygenation found in murine DSS‐induced colitis.^[^ ^17^ ^]^ Similarly, increased levels of oxygenated hemoglobin were also found by MSOT in a model with Bacteroides *fragilis*‐induced colitis.^[^ ^9^ ^]^ However, these findings are in contrast to observations of decreased mucosal oxygen saturation measured by reflectance spectrophotometry in rats^[^ ^18^ ^]^ and the hypothesis of hypoxia as a key factor in intestinal inflammation.^[^ ^19^ ^]^ In this context, our findings might be reasoned in the transmural assessment of blood oxygenation in combination with the used DSS model. Our study focused on the implementation of a reliable experimental setup capable of imaging the murine colon in vivo using OAI, however, it is limited by the assessment of a single model of colitis and the lack of an appropriate independent method to assess oxygenation in the colon wall vessels. Further investigations in different models of colitis will provide more information about the pathophysiological relationship of transmural oxygenation status and inflammation according to different disease models and progressions.

There remain technical limitations to the application of MSOT for functional assessment of early inflammation. For example, technological improvements for accurate assessment of blood oxygenation are ongoing,^[^ ^20^ ^]^ including the development of deep‐learning approaches for spectral unmixing of hemoglobin components at greater tissue depth that might improve the accuracy of quantification of oxygenation status in vivo.^[^ ^21^ ^]^ Furthermore, by applying additional wavelengths in the extended near‐infrared range of light, other chromophores such as lipids, water, and collagens might be distinguishable.^[^ ^22^ ^]^ Studies in murine models of colitis show that by this approach, inflammatory and fibrotic tissue components might be separated,^[^ ^23^ ^]^ which would be of special interest for clinical cases of stricturing Crohn`s disease, where the indication for surgery depends on this distinction.

To summarize, optoacoustic imaging is on the cusp of entering clinical practice,^[^ ^6^, ^24^ ^]^ however, in order to prove its added value, fundamental and translational studies are still necessary. Thus, TAG‐MSOT could serve as an important imaging technology for applied and translational IBD research to resolve questions about homeostatic changes and blood composition. In addition, handheld MSOT has been used to depict signs of inflammation in adult and pediatric patients with IBD,^[^ ^11^, ^12^ ^]^ while orally administered dyes were used to assess functional gastrointestinal parameters.^[^ ^6^, ^25^ ^]^ In this context, preclinical studies in mouse models afford an opportunity for biomarker validation and testing therapeutics, toward future clinical assessment and implementation.

## 4 Experimental Section

In accordance with the principles of the 3Rs (Replacement, Reduction, and Refinement) by Russell and Burch,^[^ ^26^ ^]^ all animals used in this study were also used to introduce TAG‐RSOM imaging.^[^ ^7^ ^]^ For detailed information about the fabrication of the transrectal absorber guide (TAG), please see *Buehler* et al.^[^ ^7a^ ^]^ In brief, the TAG was fabricated from a polymer tube that encloses a second smaller tube to facilitate laminar flow and the exchange of a fluid contrast agent. In contrast to TAG‐RSOM imaging, no bending forces were exerted on the TAG during MSOT imaging. This allowed the replacement of the glass capillary with a small polymer tube, reducing the impedance mismatches and subsequent acoustic artifacts.

### Animal Protocol

All animal procedures were approved by the Animal Welfare and Ethical Review Board at Cancer Research UK Cambridge Institute (project license PE12C2B96, user license I24947753), and issued under the United Kingdom Animals (Scientific Procedures) Act, 1986. For the experimental disease model, dextran sodium sulfate (DSS) (MP Biomedicals, Irvine, CA, USA) colitis was induced in female Balb/C mice (Charles River, UK). Mild colitis was induced in mice (n = 10) via oral administration of 5% DSS in drinking water for one week. Imaging was performed prior to DSS administration (day 0) and two days post‐administration (day 9) to warrant colonic inflammation. Severe colitis was induced in a second group of mice (n = 10) using a protocol of two one‐week DSS cycles. The initial cycle involved 3% DSS to induce mild inflammation, followed by a two‐week healing phase. Subsequently, 5% DSS was administered for one week to induce severe colitis. Imaging was conducted at the start (day 0 and day 21) and at the end (day 7 and day 28) of each cycle. A sham control group (n = 5) was imaged two times (day 0 and day 7) without DSS administration to evaluate the imaging protocol's effect on the animals. Health data, histological analysis, and endoscopy data o the animals used in this study were analyzed and published before in *Buehler* et al.^[^ ^7a^ ^]^

### TAG‐MSOT Imaging Protocol—Imaging System

Image acquisition was performed using a commercial multispectral optoacoustic tomography (MSOT) imaging system (MSOT inVision 256‐TF, iThera Medical GmbH, Munich, Germany) designed for preclinical research, described in detail elsewhere.^[^ ^27^ ^]^ In brief, the system employs a tunable optical parametric oscillator pumped by a Nd:YAG laser to uniformly illuminate an ≈8 mm wide ring on the imaging subject. The emitted 9‐ns long excitation pulses were absorbed by endogenous chromophores within the subject and converted into sound energy (photoacoustic effect). The generated acoustic pressure waves were measured by a concave array of 256 transducers (center frequency of 5 MHz, 60% bandwidth), providing an angular coverage of 270° and a curvature radius of 4 cm. The imaging chamber was filled with water to allow for acoustic coupling.

### TAG‐MSOT Imaging Protocol–Animal Preparation

For sedation, mice were initially administered 4% isoflurane in a gas mixture of 50% oxygen and 50% medical air. Anesthesia was maintained with a reduced isoflurane concentration of ≈1%–2% to ensure a constant respiration rate of 70–80 breaths per minute. To ensure acoustic coupling during imaging, abdominal fur was extensively removed by shearing and subsequent application of hair removal cream for 60 s. Prior to colonoscopy and the insertion of the TAG, feces were gently flushed out of the colon by squirting pre‐warmed sterile saline (35 °C) into the mouse rectum.

### TAG‐MSOT Imaging Protocol—Data Acquisition

For imaging, the TAG was inserted into the colon of the mouse, and a thin layer of transparent ultrasound gel (Aquasonic Clear, Parker Labs) was applied to the abdominal region to enhance acoustic coupling. The mouse was then placed onto a polyethylene membrane (plastic wrap). The flexible membrane was folded around an animal holder (iThera Medical GmbH) to create a watertight seal. The animal holder was subsequently transferred to the preheated (36 °C) water filled imaging chamber and connected to the MSOT system, allowing gas flow to the nose cone to maintain anesthesia. The animal holder was centered in the XY‐plane and moved along the Z‐axis through the transducer ring during imaging. Two scans were conducted using nine wavelengths (700, 730, 750, 760, 770, 800, 820, 840, and 850 nm), averaging 10 frames per wavelength at each Z‐position/slice (scan rate of 10 Hz). During the first scan, the TAG was filled with water. With a sampling rate of one slice every 0.5 mm along the Z‐axis, the acquisition time for an abdominal region was ≈16 min. For the second scan, the water inside the TAG was replaced with contrast agent (pure Indian ink, Video S2), and with a sampling rate of 2 mm the acquisition time was reduced to ≈4 min.

### TAG‐MSOT Imaging Protocol—Data Processing

First, for each slice of a contrast‐filled TAG scan, an individual speed of sound (SOS) was selected for the reconstruction. To obtain optimal results when measuring the adjacent colon wall, the SOS was set so that the TAG formed a circular shape with a diameter of 3 mm. The viewMSOT software package (version 4.0, iThera Medical GmbH, Munich, Germany) was used for model‐based reconstruction (resolution: 75 µm) and spectral unmixing of deoxygenated (HbR) and oxygenated hemoglobin (HbO_2_). Total hemoglobin (HbT = HbR + HbO_2_) and MSOT‐measured oxygen saturation (mSO_2_ = HbO_2_ / HbT) were calculated based on unmixed hemoglobin signals. A region of interest (ROI) was defined by two concentric circles with diameters of 3 mm (TAG) and 3.4 mm (TAG & colon tissue). For quantification, the SOS and ROI were maintained across the corresponding slices of water‐filled TAG images. The mean pixel intensities within the ROIs of five successive slices along the Z‐axis (step size 2 mm) were averaged to obtain signal intensities for the single wavelength measurement (800 nm) and hemoglobin‐derived parameters.

### TAG‐MSOT Imaging Protocol–3D Data Visualization

Scans with water‐filled TAG were performed at an increased sampling rate of one slice every 0.5 mm along the Z‐axis to facilitate high‐resolution 3D rendering using 3Dscript software.^[^ ^28^ ^]^ For this purpose, model‐based reconstruction was applied using a uniform speed‐of‐sound throughout the entire volume. To visualize the colon wall, cropping was approximated by a cylinder matching the size and orientation of the TAG.

### Statistics

Distribution of the data was tested using the Shapiro‐Wilk test. Subsequently, one‐way ANOVA was applied to compare normally distributed data, and the Kruskal‐Wallis test was used for non‐normal distributed data. Multiple comparisons were corrected for by applying Šidák's and Dunn's test, respectively. Correlations were assessed using Spearman correlation. All statistical analyses were performed with GraphPad Prism software (Version 9, GraphPad Software, Inc., San Diego, CA, USA). Results were expressed as mean ± standard deviation. Two‐tailed P‐values < 0.05 were considered statistically significant.

## Conflict of Interest

M.J.W., F.K., and A.P.R. are shared patent holders with iThera medical GmbH (Munich, Germany) on an optoacoustic imaging system/software, similar to the one described in the study.

## Author Contributions

S.E.B. and A.P.R. contributed eqaully to this work. A.B., F.K., S.E.B., and A.P.R. designed the study. A.B. developed the TAG. A.B., M.E.O., and E.L.B. performed all animal experiments. A.B. performed all imaging analyses. M.E. scored the histopathology data. F.K. scored the endoscopy data. A.B., F.K., S.E.B., and A.P.R. analyzed the data. A.B., F.W., M.J.W., F.K., S.E.B., and A.P.R. interpreted the data. A.B. and A.P.R. wrote the first draft of the manuscript. The manuscript was critically reviewed by all authors.

## Supporting information



Supporting Information

Supplemental Video 1

Supplemental Video 2

## Data Availability

The data that support the findings of this study are openly available in Research data supporting: “Guided Multispectral Optoacoustic Tomography for 3D Imaging of the Murine Colon” at https://doi.org/10.17863/CAM.114332.
