# Supplementary material for: Guided Multispectral Optoacoustic Tomography for 3D Imaging of the Murine Colon
Source: Adv Sci (Weinh). 2025 Jan 21;12(10):2413434. doi: 10.1002/advs.202413434 (PMC11905093; doi:10.1002/advs.202413434)
Supplement: Supplementary file 1 — Supporting Information [file ADVS-12-2413434-s003.docx]

Supporting Information

­­

**Guided Multispectral Optoacoustic Tomography for Three-Dimensional Imaging of the Murine Colon**

*Adrian Buehler, Emma L. Brown, Markus Eckstein, Oana-Maria Thoma, Felix Wachter, Henriette Mandelbaum, Petra Ludwig, Merle Claßen, Mariam-Eleni Oraiopoulou, Ulrich Rother, Markus F. Neurath, Joachim Woelfle, Maximilian J. Waldner, Oliver Friedrich, Ferdinand Knieling, Sarah E. Bohndiek, and Adrian P. Regensburger*


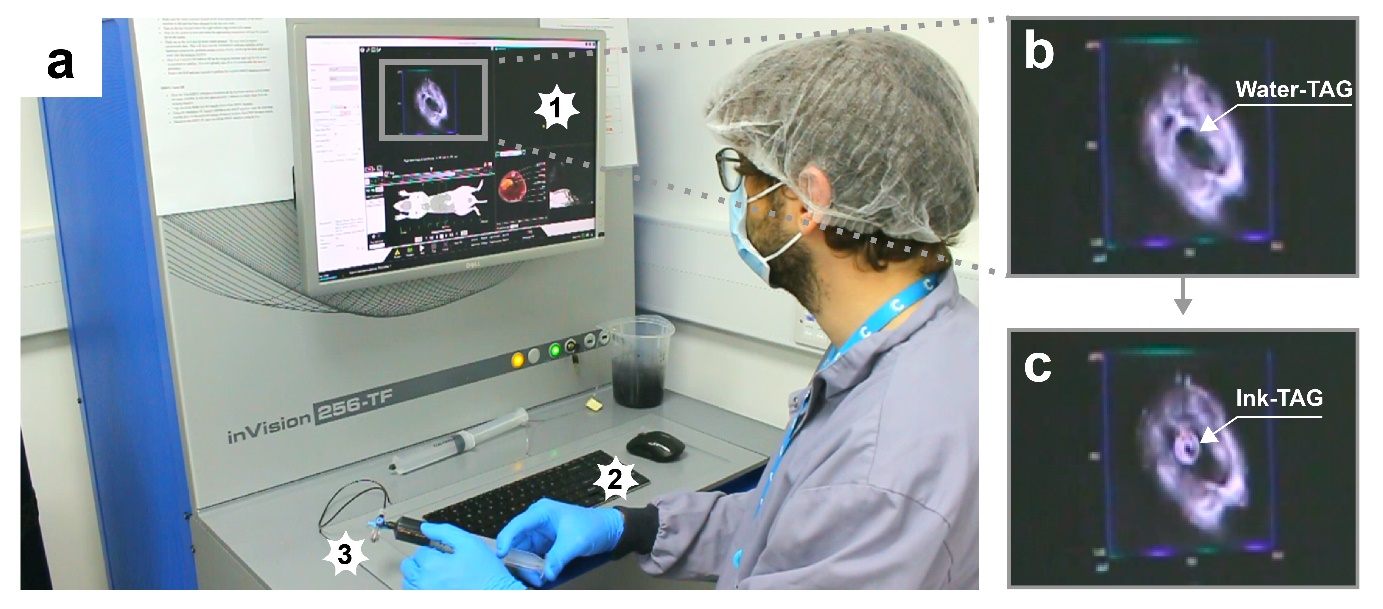


**Figure S1.**

Transrectal absorber guide Multispectral optoacoustic tomography (MSOT). **a** The multispectral optoacoustic tomography (MSOT inVision 256-TF, iThera Medical GmbH, Munich, Germany) system used in this study features a monitor (**1**) for control of all imaging parameters and displaying a live optoacoustic image of the animal. The animal holder is placed inside the water tank, and the sliding lid (**2**) is closed to ensure safe operation. Contrast was changed (**3**) between scans by accessing the TAG via flexible tubes that lead inside the imaging chamber. **b**-**c** Successful change of contrast was validated using the live view.


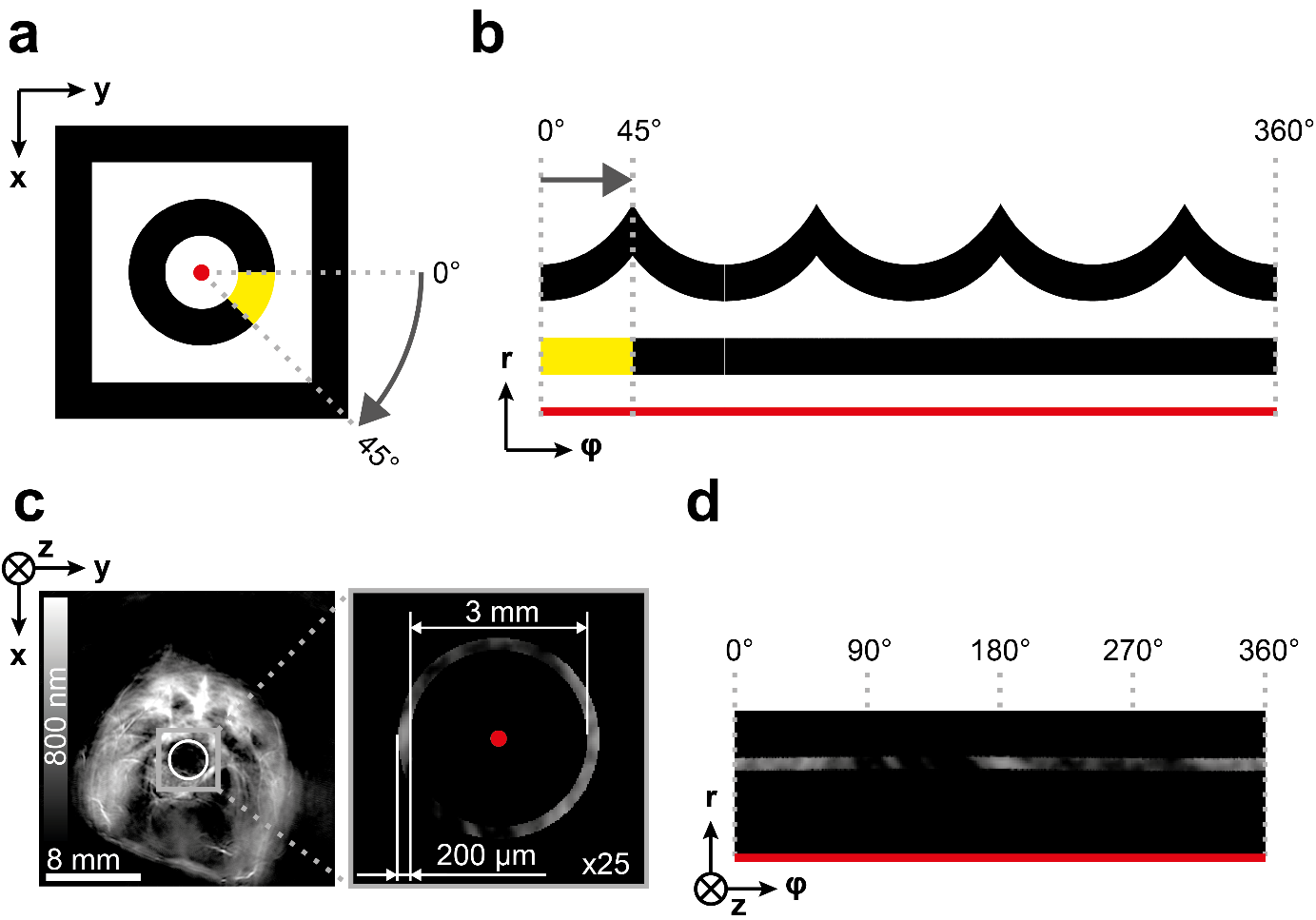


**Figure S2.**

Polar transformation. The exemplary 2D image shown in **a** depicts a central dot, a circle and a square. After polar transformation using the dot as the transformation center, changes in the characteristic color and shape features can be seen in **b**. For enhanced visualization of TAG-MSOT measurements, the segmented colon walls (**c**) are polar-transformed (**d**) to enable radial projections over all slices as shown in **Figure 2** and **Figure S3-S6**.


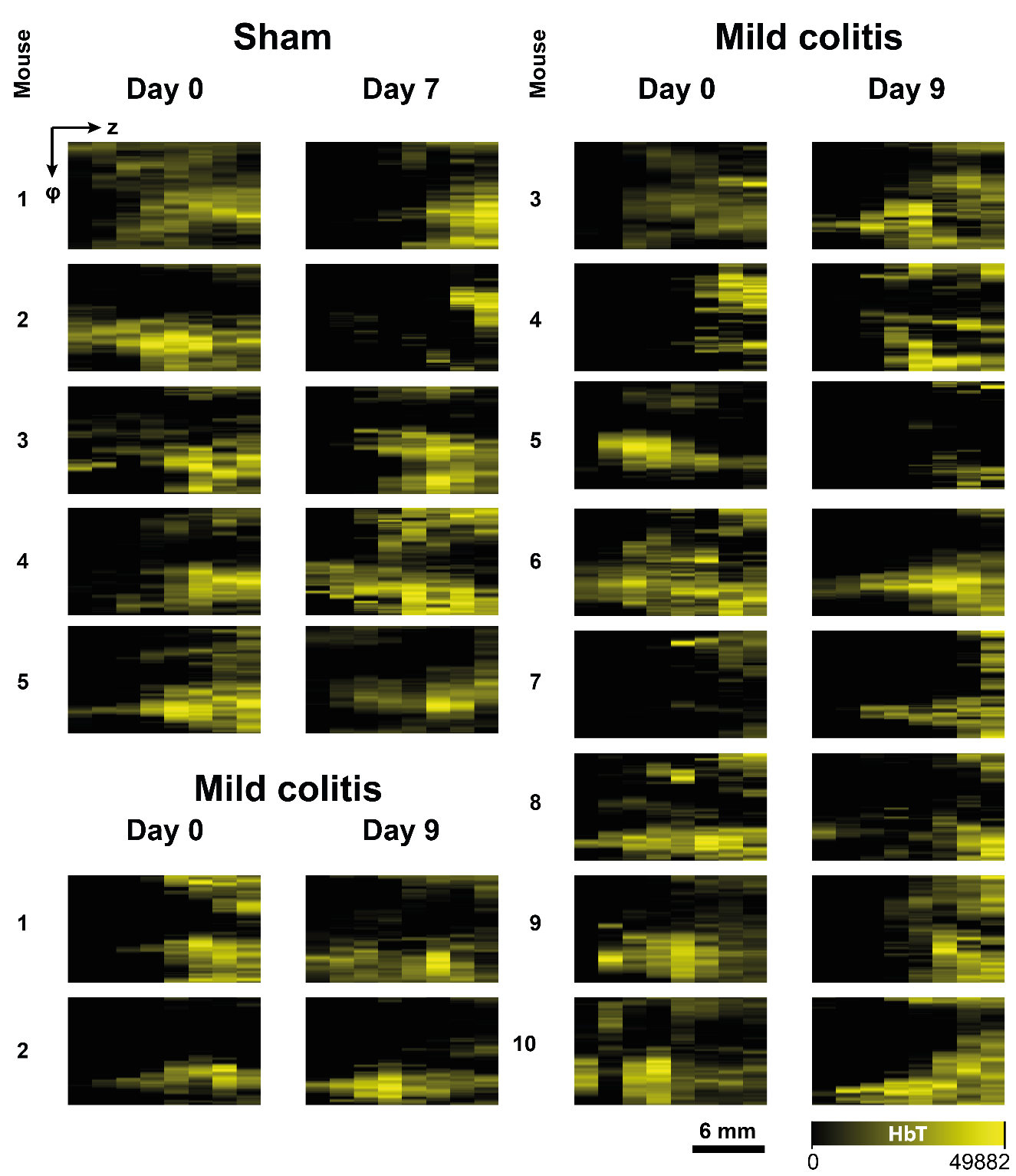


**Figure S3.**

Total hemoglobin (HbT) measured by TAG-MSOT in the murine colon wall during mild colitis. Individual 2D radial projections are calculated as described in **Figure S2** and analyzed as described in **Figure 2**.


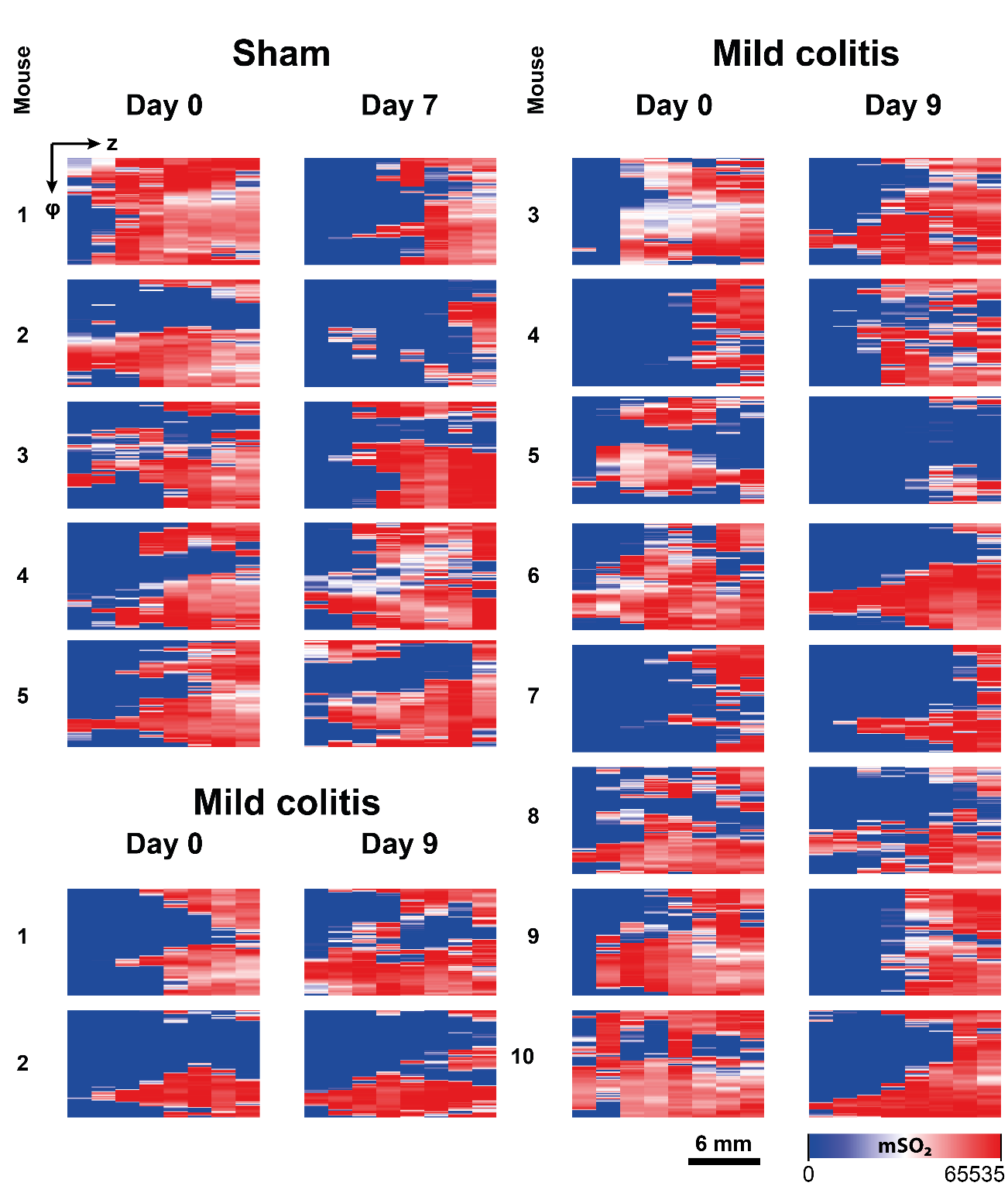


**Figure S4.**

Blood oxygenation (mSO_2_) of the murine colon wall during mild colitis. Individual 2D radial projections are calculated as described in **Figure S2** and analyzed as described in **Figure 2**.


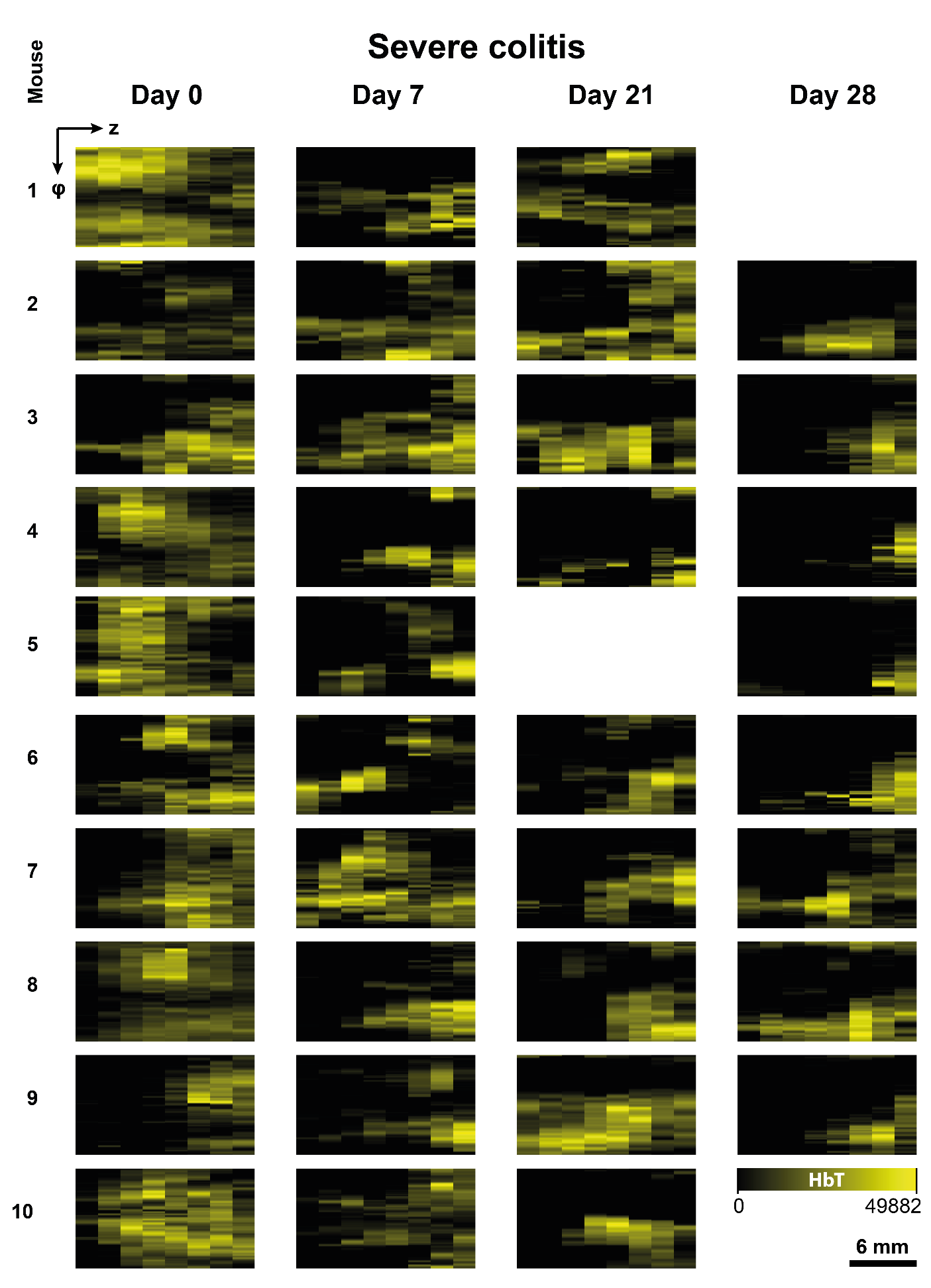


**Figure S5.**

Hemoglobin measured by TAG-MSOT in the murine colon wall during severe colitis. Individual 2D radial projections are calculated as described in **Figure S2** and analyzed as described in **Figure 2**.


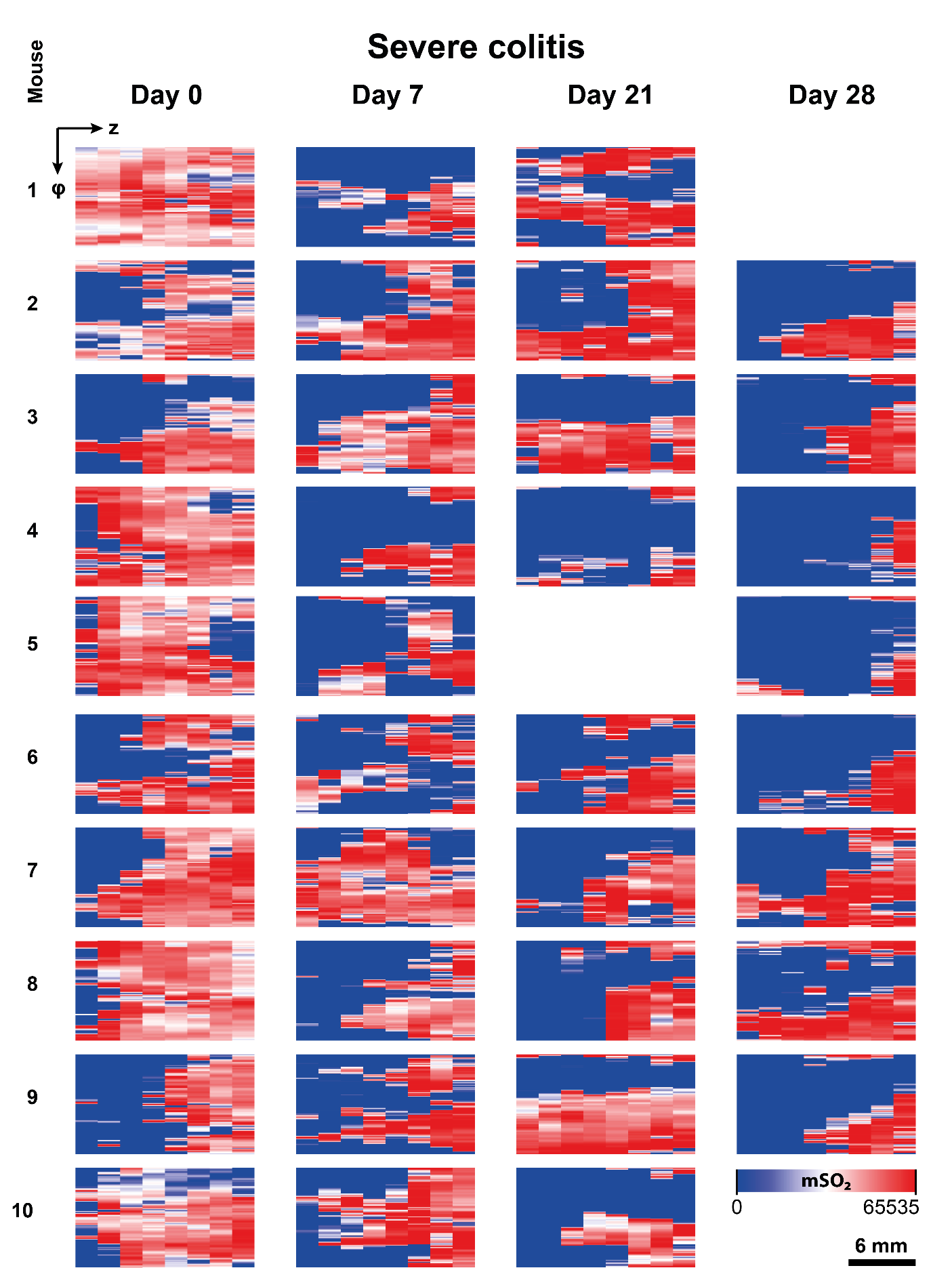


**Figure S6.**

Blood oxygenation of the murine colon wall during severe colitis. Individual 2D radial projections are calculated as described in **Figure S2** and analyzed as described in **Figure 2**.


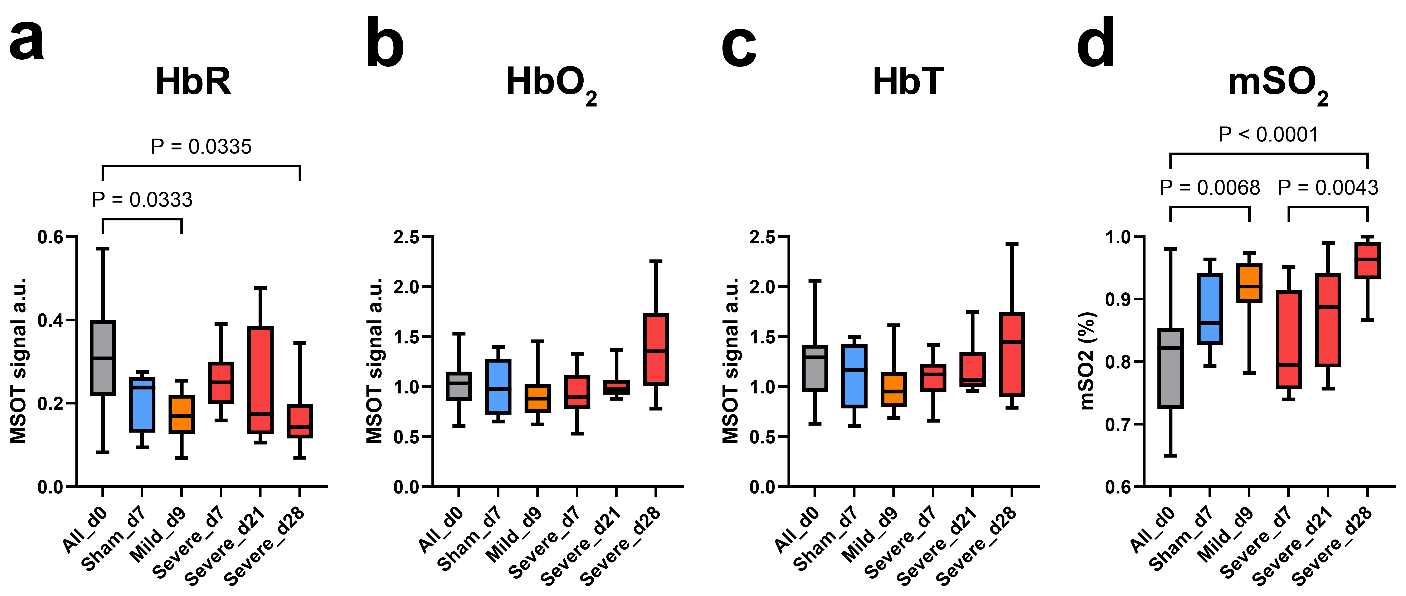


**Figure S7.**

Overview of TAG-MSOT hemoglobin parameters of all studies. In this figure, the measured TAG-MSOT hemoglobin parameters of the sham control (n=5), mild (n=10) and severe colitis (n=10) study are compared. Here, all TAG-MSOT scans of healthy mice are plotted as the timepoint All_d0 (n=25). Deoxygenated hemoglobin (HbR) (**a**) and oxygenated hemoglobin (HbO_2_) (**b**) were unmixed, and the total hemoglobin signal (HbT) (**c**) and blood saturation (mSO_2_) (**d**) were calculated. The data is presented as box plots (showing the median, 25th and 75th percentiles, with whiskers representing the minimum and maximum values). Statistical analyses were performed using the Kruskal-Wallis test with Dunn’s correction (**a**-**c**) and one-way ANOVA with Šidák’s correction (**d**). Non-significant results (P > 0.05) are not displayed in the graphs.
